# Nonoperative management of rectal cancer

**DOI:** 10.3389/fonc.2024.1477510

**Published:** 2024-12-06

**Authors:** Hannah Williams, Christina Lee, Julio Garcia-Aguilar

**Affiliations:** Colorectal Service, Department of Surgery, Memorial Sloan Kettering Cancer Center, New York, NY, United States

**Keywords:** watch-and-wait, locally advanced rectal cancer, total neoadjuvant therapy, local regrowth, nonoperative management

## Abstract

The management of locally advanced rectal cancer has changed drastically in the last few decades due to improved surgical techniques, development of multimodal treatment approaches and the introduction of a watch and wait (WW) strategy. For patients with a complete response to neoadjuvant treatment, WW offers an opportunity to avoid the morbidity associated with total mesorectal excision in favor of organ preservation. Despite growing interest in WW, prospective data on the safety and efficacy of nonoperative management are limited. Challenges remain in optimizing multimodal treatment regimens to maximize tumor regression and in improving the accuracy of patient selection for WW. This review summarizes the history of treatment for rectal cancer and the development of a WW strategy. It also provides an overview of clinical considerations for patients interested in nonoperative management, including restaging strategies, WW selection criteria, surveillance protocols and long-term oncologic outcomes.

## 1 Introduction

Colorectal cancer is one of the most common malignancies in the United States, with a third of cases located in the rectum (1). The management of rectal cancer has changed drastically in the last few decades due to improved surgical techniques and the development of multimodal treatment approaches. Until recently, the standard of care for locally advanced rectal cancer (LARC) included neoadjuvant chemoradiation and total mesorectal excision (TME) followed by adjuvant chemotherapy. With this approach, up to 18% of patients had complete eradication of tumor, or a pathologic complete response (pCR), on surgical specimen (2, 3). Studies of patients with a pCR demonstrated excellent long-term outcomes, with 5-year overall survival and disease-free survival rates approaching 90% and 87%, respectively (4–6).

This excellent prognosis raised the question of whether patients with a pCR gained any oncologic benefit from surgical resection. In 2004, Habr-Gama et al. reported outcomes from the first group of highly selected patients enrolled in a watch and wait (WW) surveillance program (7). The safety and efficacy of this approach relied on accurately identifying a complete response using a clinical assessment in place of histologic confirmation of a pCR. WW offered the promise of organ preservation and improved quality of life by eliminating the long-term functional deficits associated with TME. Since Habr-Gama et al’s landmark paper, an extensive volume of literature has demonstrated that WW is a viable treatment strategy that does not compromise long-term oncologic outcomes (8–11). However, challenges remain in accurately identifying patients with complete eradication of disease and in optimizing multimodal treatment regimens to maximize tumor response.

In this article, we review the history of multimodal treatment for LARC and the development of a WW approach. Next, we summarize the restaging process and assess the selection criteria for WW, surveillance strategies and long-term outcomes.

## 2 Common terms and definitions in watch and wait

Before exploring the history of WW and its current applications, it is important to first define several common terms used in the literature. LARC includes stage II (T3/T4N0) or III (T1-4N1-2) rectal tumors. A pCR is the gold-standard for confirming eradication of tumor and corresponds to ypT0N0 on TME specimen. In patients considered for nonoperative management, a clinical complete response (cCR) is used as a surrogate marker for a pCR. Patients with a cCR have no evidence of residual disease on restaging flexible sigmoidoscopy, MRI or clinical exam after completing neoadjuvant treatment. Current assessment measures cannot predict a pCR with absolute accuracy, and some of the patients with a cCR subsequently develop local regrowth (12). Patients who continue to have no evidence of residual disease for two years after restaging are considered sustained cCRs (s-cCR). Organ preservation refers to the desired outcome whereby a patient with a s-cCR successfully preserves the rectum with a WW strategy.

A near complete response (nCR) is a relatively recent addition to the clinical response ranking system. These patients have minor abnormalities at restaging, which may resolve into a s-cCR with a longer period of observation. However, some patients with a nCR never achieve a cCR. The exact criteria and management guidelines for a nCR are poorly described, and subsequent sections will discuss this topic in more detail. An incomplete clinical response (iCR) refers to patients who have evidence of residual tumor at restaging. These patients are not candidates for WW and should proceed to TME. In addition to clinical response, other patient factors may influence recommendation for surgical resection, including baseline fecal incontinence and anticipated difficulty adhering to a strict WW surveillance schedule.

**Figure 1:**
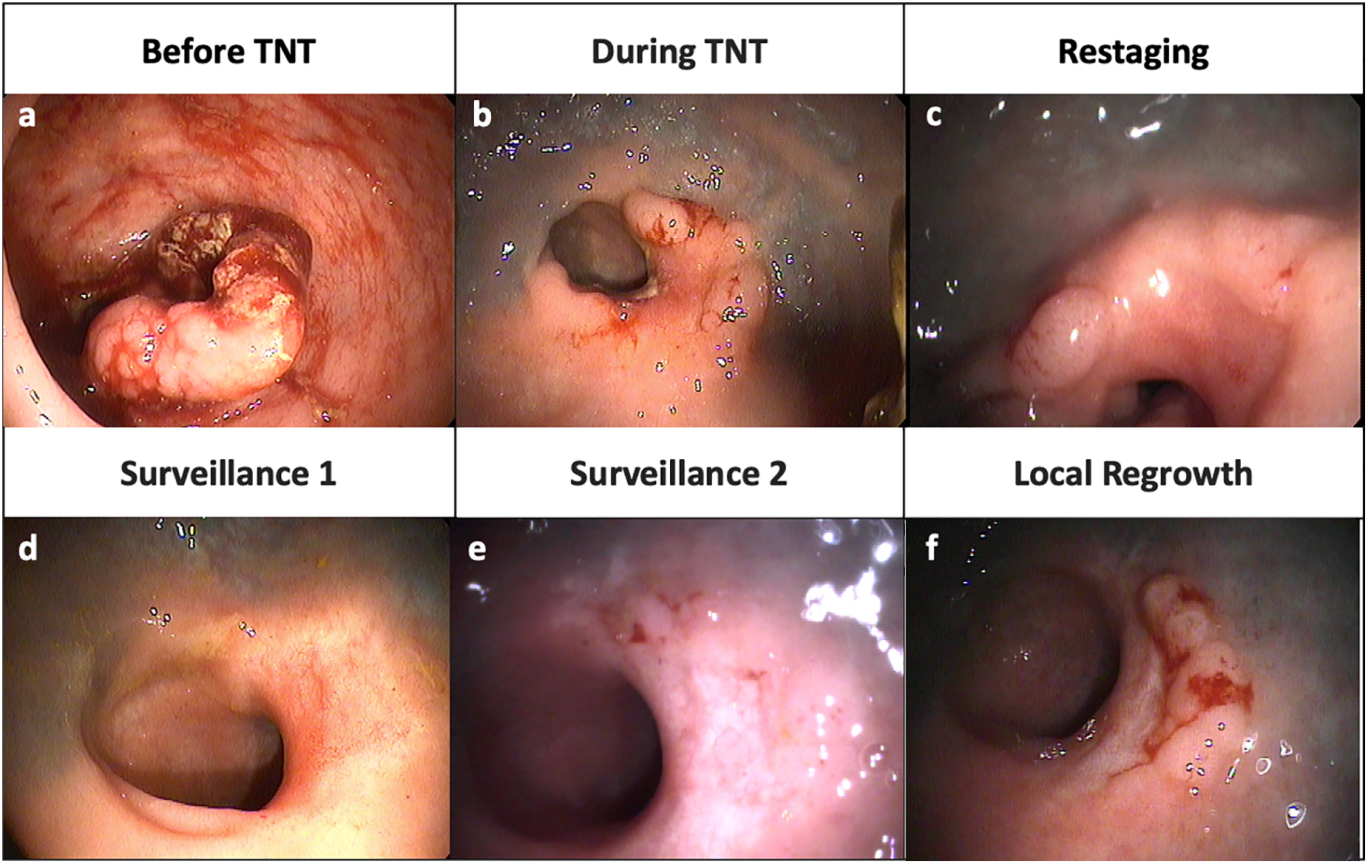
Representative endoscopic images from a patient with local regrowth. **(A)** Tumor at diagnosis **(B)** Shrinkage of tumor during TNT **(C)** Nodularity and mild mucosal abnormality at restaging **(D)** A flat, white scar and telangiectasia consistent with a clinical complete response **(E)** Re-development of some mild mucosal abnormalities **(F)** Obvious local tumor regrowth along right lateral wall.

Local regrowth refers to disease reappearance at the site of the treated tumor in patients managed with a WW strategy (**Figure 1**). Local regrowth can occur in the rectal wall, mesorectum or within the lateral internal iliac or obturator lymph nodes (13). The majority of patients with local regrowth undergo successful salvage TME with R0 margins (11, 14, 15). This term should be distinguished from local recurrence (LR), which refers to non-salvageable local regrowth or recurrent cancer in the pelvis following a curative resection.

## 3 Historical management of locally advanced rectal cancer

Until the 1980s, the effectiveness of surgical resection for LARC remained limited with 15-40% of patients developing pelvic recurrence, significant rates of postoperative mortality, and 5-year overall survival rates of 50-70% (16–19). Two innovations- preoperative (chemo)radiation and TME– greatly improved locoregional outcomes. Heald et al. first reported their institution’s experience with TME in 1986, noting that the technique greatly reduced rates of LR and improved overall survival (20). Subsequent pathologic studies reinforced the importance of TME for local disease control by demonstrating that specimens with a violated mesorectal plane were more likely to have positive circumferential resection margins or an inadequate lymph node harvest (21, 22). During the same period, the Swedish Rectal Cancer Trial found that patients who received preoperative short course radiotherapy had significantly lower rates of LR compared to patients who proceeded directly to surgery (23–25). However, the study was criticized for the use of non-standardized surgical techniques, which may explain the higher-than-expected rates of LR in the surgery only group. The subsequent Dutch TME trial demonstrated that preoperative radiation followed by TME reduced LR by 50% without any improvement in overall survival compared to TME alone (26, 27). The CAO/ARO/AIO-94 trial addressed the question of whether radiation should be delivered before or after surgery, with results showing that the preoperative chemoradiation arm had better compliance, less toxicity, increased tumor downstaging and lower rates of LR (28).

Two types of preoperative radiation regimens are available. These include short-course radiotherapy (SCRT) delivered as 25 Gy in 5 fractions and long-course radiotherapy (LCRT) delivered as 45-54 Gy in 25-30 fractions with sensitizing fluorouracil or capecitabine. While LCRT is favored by most providers in the United States, SCRT offers several advantages such as more efficient resource utilization, lower cost to the healthcare system and shorter treatment duration with fewer hospital visits for patients (29). Multiple randomized control trials have compared LCRT to SCRT and found no differences in terms of toxicity, local tumor control, survival or quality of life (30–32).

Simultaneous with the attempts to improve local tumor control, trials explored the use of systemic chemotherapy to reduce the risk of distant metastases, which remained a major driver of mortality in patients with LARC (33, 34). The results of these studies are mixed, with some showing a survival benefit (35, 36), while others found no improvement in oncologic outcomes (37–39). Additionally, these trials demonstrated poor adherence to adjuvant chemotherapy, with nearly 50% of patients declining treatment or receiving less than the recommended dose (33, 40).

Poor tolerance of adjuvant chemotherapy, along with the realization that tumor response to neoadjuvant chemoradiation was time-dependent, generated interest in total neoadjuvant therapy (TNT) (3). TNT delivers chemotherapy preoperatively, either as induction chemotherapy followed by chemoradiation (INCT-CRT), or as chemoradiation followed by consolidation chemotherapy (CRT-CNCT). The most common systemic chemotherapy regimens involve either eight cycles of FOLFOX (leucovorin, fluorouracil, oxaliplatin) or five cycles of CapeOx (capecitabine, oxaliplatin).

This multimodal approach offers several potential advantages, including increased tumor downstaging, earlier introduction of systemic chemotherapy to combat micrometasases, better compliance and decreased time with a temporary ostomy postoperatively (40–42). While approximately 18% of patients receiving neoadjuvant chemoradiation have a pCR, studies evaluating TNT regimens have demonstrated rates closer to 40% (3, 41, 43). Additionally, randomized control trials have shown that when compared to the standard of care (neoadjuvant chemoradiation, TME and adjuvant chemotherapy), patients receiving TNT have better disease-free survival and lower rates of distant metastases (3, 43–45). As a result, the National Comprehensive Cancer Network (NCCN) now recommends TNT as first line treatment for LARC (46).

### 3.1 Multimodal treatment for LARC: ongoing controversies and future directions

Many TNT sequences have been proposed, with studies incorporating different neoadjuvant radiation regimens (SCRT vs LCRT) accompanied by variable doses of systemic chemotherapy (8, 43, 44, 47). Our understanding of the optimal dosing required to obtain adequate local and distant disease control while minimizing toxicity remains poor. Furthermore, it is unclear whether certain patient subgroups benefit more from one TNT regimen over another.

Randomized control trials have not yet directly compared SCRT and LCRT as part of a TNT protocol. The RAPIDO, STELLAR and POLISH II trials all randomized patients to a TNT arm consisting of SCRT, consolidation chemotherapy and TME, or to a standard of care arm (LCRT, TME, ± adjuvant chemotherapy) (43, 48, 49). The STELLAR and POLISH II trials reported equivalent oncologic outcomes for each treatment regimen (48, 49) while the RAPDIO trial found a higher incidence of locoregional failure among patients randomized to receive SCRT followed by consolidation chemotherapy (50). The higher rate of LR in the RAPIDO trial’s experimental arm is difficult to explain. It is possible that the longer interval between radiation and TME in the experimental arm led to increased pelvic fibrosis and therefore compromised the quality of surgical resection. Whether a TNT regimen with LCRT offers better locoregional control than a SCRT-based TNT sequence remains unclear, as none of the above trials offered upfront chemotherapy in the LCRT arm. Data from the ongoing ACO/ARO/AIO-18.1 trial will provide the first opportunity to directly compare outcomes between patients randomized to receive either SCRT or LRCT followed by consolidation chemotherapy (51).

There is growing interest in exploring treatment de-escalation strategies to reduce exposing patients to unnecessary toxicity. For patients with high rectal tumors, the PROSPECT trial has demonstrated that induction FOLFOX with selective chemoradiation is non-inferior to LCRT, TME and optional adjuvant chemotherapy (52). Nearly 90% of patients randomized to the induction FOLFOX arm were able to proceed directly to surgery, indicating that most patients with high rectal tumors can safely avoid neoadjuvant chemoradiation (52). However, these findings are not generalizable to tumors with aggressive baseline features or to patients pursuing nonoperative management. Specific neoadjuvant treatment considerations for patients interested in WW are discussed in further detail below.

## 4 Development of a watch and wait approach to rectal cancer

Janeway first described the successful treatment of rectal tumors with contact radiation and implantation of radioactive seeds in 1917 (53). While this approach remained a cornerstone of rectal cancer management for decades, it fell out of favor with advances in perioperative safety and improvements in surgical outcomes (54). In the modern era, Habr-Gama has pioneered the study of non-operative management for tumors with a complete response to neoadjuvant chemoradiation (7). Her landmark paper from 2004 compared outcomes between 71 WW patients and 22 patients with a pCR after surgical resection. Patients were evaluated 8 weeks after completion of chemoradiation using endoscopy, digital rectal exam (DRE) and CT imaging. Patients with a cCR followed a strict surveillance schedule, including monthly clinical and endoscopic exams, as well as pelvic CT scans every 6 months for the first year. The surveillance interval increased to 2 and 6 months during the second and third years, respectively. With a mean follow-up of 57.3 months, two (2.8%) WW patients developed salvageable local regrowths and three (4.2%) developed distant metastases. Five-year disease-free survival was 92% and did not differ significantly from patients with a pCR (7).

Habr-Gama et al’s findings suggested that a clinical restaging assessment could detect a complete response and that patients with a cCR could be safely monitored using a strict surveillance protocol. Subsequent studies validated these findings, repeatedly showing that patients who achieved a s-cCR had oncologic outcomes comparable to those with a pCR (9–11, 55–58). Nevertheless, WW remained confined to specialized academic centers, with many providers hesitant to adopt the approach in the absence of data from a randomized control trial.

The Organ Preservation in Rectal Adenocarcinoma (OPRA) Trial was the first randomized phase II trial to evaluate long-term oncologic outcomes in patients offered WW (8). Participants with LARC were randomized to receive either INCT-CRT or CRT-CNCT and were restaged 8 ± 4 weeks after completion of neoadjuvant treatment using flexible sigmoidoscopy, DRE and MRI. Long-term follow-up demonstrated that nearly half (46.7%) of the enrolled patients achieved organ preservation (59). Oncologic outcomes did not differ compared to historical controls that underwent TME (8, 59).

## 5 Watch and wait: approach to patient management

### 5.1 Diagnosis and neoadjuvant treatment

Patients with newly diagnosed rectal cancer should obtain a full colonoscopy, mismatch repair (MMR) testing, pelvic MRI, CT chest, abdomen, pelvis and a baseline serum carcinoembryonic antigen (CEA) level to complete primary staging (46). MMR deficient tumors are highly responsive to immune checkpoint inhibitors (60). While a more robust discussion of immunotherapy’s long-term efficacy and safety in WW patients lies outside the scope of this review, it is important to note that this subgroup has alternative treatment options available. Among patients with MMR proficient tumors, those with stage II or III rectal cancer are potentially eligible for WW.

Several baseline tumor characteristics, including tumor length, cN+ disease, extramural venous invasion (EMVI) and mesorectal fascia involvement may be associated with a higher risk of residual tumor after TNT and/or worse oncologic outcomes (45, 61–63). However, the data on this topic can be contradictory, and further research is needed to better delineate the prognostic significance of baseline tumor features in patients treated with TNT. Presence of these characteristics should not preclude patients from pursuing a selective WW strategy.

Multidisciplinary management including colorectal surgery, medical oncology, radiology, pathology and radiation oncology is crucial for optimizing patient outcomes. Although many variations in multimodal treatment exist, we will focus on TNT, as it is the standard of care. Patients potentially eligible for nonoperative management can receive either INCT-CRT or CRT-CNCT. No consensus exists regarding the superiority of INCT-CRT versus CRT-CNCT. The CAO/ARO/AIO-12 and OPRA trials have shown a higher incidence of complete response among patients treated with CRT-CNCT, suggesting that this may be the preferred sequence for those interested in organ preservation (8, 47, 59). However, neither trial demonstrated a significant difference between the two treatment regimens in terms of oncologic outcomes or treatment-related toxicities (47, 59, 64, 65). The duration of systemic chemotherapy and type of radiation (SCRT vs. LCRT) needed to optimize a selective WW strategy remain unknown and will require investigation with randomized control trials.

### 5.2 Components of the restaging exam

The safety and success of a WW strategy relies on accurately selecting patients with a complete response to neoadjuvant treatment, while also correctly identifying those with residual tumor who require TME. All modalities of the restaging assessment, which typically consists of DRE, flexible sigmoidoscopy, and pelvic MRI, cannot predict a complete response with absolute certainty (12). Endoscopic biopsies are not routinely recommended, as they have a high false negative rate and cannot be relied upon to guide patient management (66). Providers should use information from all three components of the restaging exam to categorize patients as a cCR, nCR or iCR. **Table 1** presents the DRE, endoscopic and MRI features developed through expert consensus for the OPRA trial to stratify response at the end of TNT.

**Table 1:** OPRA trial clinical response criteria.

	Clinical Complete Response	Near Complete Clinical Response	Incomplete Clinical Response
DRE	• Normal	• Smooth induration	• Palpable tumor
Endoscopy	• Flat, white scar • Telangiectasias • No ulceration • No nodularity	• Superficial ulceration • Small nodules • Irregular mucosa • Mild erythema of the scar	• Visible tumor
MRI	• Only dark T2 signal • Invisible or very few lymph nodes <5mm in SAD • Absent restricted diffusion	• Mostly dark T2 signal with 1-2 foci of intermediate T2 signal • Partially regressed lymph nodes (≥ 5mm in SAD) • Significant regression of restricted diffusion	• More intermediate than dark T2 signal • Persistently enlarged lymph nodes • Persistent restricted diffusion

DRE, digital rectal exam; MRI, magnetic resonance imaging; SAD, short axis diameter.

#### 5.2.1 Digital rectal exam

Response on DRE can be divided into a completely normal examination (cCR), induration or fullness (nCR) or palpable tumor (iCR). While this is an essential component of the restaging exam, it is important to note that DREs have poor accuracy in determining a complete response (67). The physician should always note whether the lesion is palpable at baseline, as some high rectal tumors cannot be reached on digital exam.

#### 5.2.2 Endoscopic exam

Endoscopy provides crucial information about a tumor’s luminal response to treatment. The standardized definition of an endoscopic cCR includes a flat white scar, telangiectasias and absence of ulceration, nodularity or other mucosal abnormalities (8, 68). Features of a nCR may include superficial ulceration, minor mucosal abnormalities, erythema of the scar or nodularity (8). Finally, an endoscopic iCR is defined as obvious residual tumor on exam.

#### 5.2.3 Pelvic MRI

MRI provides information about the status of the bowel wall, mesorectum and regional lymph nodes. While no standardized criteria of clinical response grade by restaging MRI exist, general consensus is as follows. A cCR is defined as complete absence of residual tumor, exclusively dark T2 signal, no restricted diffusion on diffusion-weighted imaging (DWI) and very small or invisible lymph nodes. A nCR includes mostly dark T2 signal, significant regression of restricted diffusion on DWI and partial regression of visible lymph nodes during TNT. An iCR is defined as multiple regions of intermediate T2 signal, persistent restricted diffusion on DWI and persistently enlarged lymph nodes (8, 69). In addition to these features, EMVI and abnormal lymph node morphology may also indicate increased likelihood of residual disease (70–73).

### 5.3 Timing of the restaging assessment

Tumor response to neoadjuvant treatment is time-dependent, and multiple studies have demonstrated that rates of pCR increase in conjunction with time from end of neoadjuvant treatment (74–78). Hesitancy about leaving potentially untreated disease *in situ*, as well as concerns that severe radiation-induced fibrosis could complicate surgical resection, made surgeons reluctant to delay TME beyond 6 weeks (3, 79). With the exception of the GRECCAR-6 trial, all other studies on this topic have demonstrated similar rates of postoperative morbidity and comparable oncologic outcomes among patients taken to TME at a ≥ 6 week interval from end of neoadjuvant treatment (76, 78, 80–82). When applied in the setting of WW, allowing more time for tumor regression increases the proportion of patients with a cCR at restaging. While it is now standard to perform the restaging assessment approximately 8 weeks after end of neoadjuvant therapy, Habr-Gama et al. have demonstrated that tumors take a median of 18.7 weeks to meet the strict criteria of a cCR (83).

### 5.4 Diagnostic accuracy and limitations of the restaging assessment

The restaging assessment cannot predict a cCR with perfect accuracy. With the exception of MMR deficient tumors, which are targetable with immunotherapy, no genetic markers exist that predict response to neoadjuvant treatment (60). Of the three components that make up the restaging exam, endoscopy is the most accurate. It has a reported sensitivity of 0.53, specificity of 0.92-0.97 and accuracy of 0.80-0.89 for detecting a complete response (12, 84–86). Analyses of specific restaging endoscopic features have found a flat, white scar predictive of a complete response (87, 88). Mild mucosal abnormalities are responsible for most patients incorrectly classified as an iCR (87, 89).

The diagnostic performance for restaging MRI is slightly worse, with a reported sensitivity of 0.50-0.65, specificity of 0.63-0.91 and accuracy of 0.64-0.79 for predicting T stage, and an accuracy of 0.60-0.79 for detecting positive lymph nodes (12, 69, 70, 90–92). Radiation-induced fibrosis, bowel wall edema and desmoplastic reaction make interpreting post-treatment MRIs complex, as residual tumor can be indistinguishable from the effects of neoadjuvant treatment (93, 94). Multiple methods have been investigated to improve the diagnostic performance of restaging MRI. Adding DWI to conventional sequences improves accuracy, but this technique is subject to high rates of interobserver variability (90, 95–97). Other avenues of investigation include using dynamic contract enhanced MRI, magnetic resonance tumor regression grade (mrTRG) and MR volumetry (98).

A single prospective study of 50 patients by Maas et al. evaluated the combined performance of restaging endoscopy, DRE and MRI (12). The authors found that the combined results yielded a positive post-test probability of 98% for detecting a complete response when all modalities indicated a cCR. Conversely, when all tests indicated a non-complete response, there was a negative post-test probability of 15% that the patient would have a pCR (12). Improving the diagnostic performance of the restaging exam remains one of the greatest challenges to expanding WW. Very little information exists on the accuracy of the combined restaging assessment, or the prognostic implications in situations where the clinical response grade differs between endoscopy and MRI.

### 5.5 Which patients should be allowed to enter watch and wait surveillance?

Due to concerns about the oncologic safety of WW, eligibility for nonoperative management was initially restricted to patients with a cCR at restaging (68). According to these criteria, any patient with even mild abnormalities by endoscopy or MRI proceeded to TME. Multiple studies have since shown that a large proportion of patients who did not meet this narrow definition of a cCR had a pCR by the time of surgery (87, 99–101). This observation, along with data demonstrating the oncologic safety of delaying surgery by several weeks, spurred interest in expanding eligibility for nonoperative management. Several studies have allowed patients with a nCR to enter WW surveillance (8, 102, 103). In the OPRA trial, nearly 40% of patients with a nCR developed a s-cCR with nonoperative management (59). The remainder went on to develop signs of residual rectal tumor, highlighting the importance of short-interval reassessment in this high-risk group.

Of note, several studies have explored performing local excisions for patients with a nCR at restaging (103, 104). Endoscopic mucosal excision may be appropriate for some patients with a small area suspicious for residual tumor and an endoscopic biopsy showing only high-grade dysplasia. The role of full thickness local excision for invasive residual tumor is controversial. While there is evidence that some residual tumor limited to the submucosa or muscularis mucosa could potentially be cured by a full-thickness local excision, the distribution of the residual cancer cells throughout the bowel wall in patients with LARC treated with TNT is difficult to predict (105, 106). In these cases, a local excision may be oncologically insufficient. Furthermore, a failed local excision due to positive resection margins, unfavorable histology, or local recurrence, can compromise a salvage TME by violating the resection planes and/or compromising the possibility of a sphincter-saving procedure (107, 108). In addition to concerns about this approach’s oncologic safety in LARC patients, local excision leads to high rates of wound dehiscence, poor functional outcomes and worse overall quality of life compared to those managed by WW alone (102, 104, 109).

### 5.6 Surveillance

No standardized surveillance protocols for patients managed by WW exist. The OPRA trial followed patients for five years after restaging (8). Surveillance included a H&P, flexible sigmoidoscopy and CEA testing at 3- to 6-month intervals for two years and then at 6-month intervals for the remaining three years. A MR pelvis was obtained every 6 months for the first year and then annually for the remaining four years. A CT chest, abdomen, pelvis was performed annually to monitor for development of distant metastases. Patients with a nCR at restaging were often monitored with flexible sigmoidoscopy at shorter intervals (4 to 6 weeks) to confirm continued tumor regression. These patients were taken promptly to TME if the tumor stopped responding to treatment or progressed during the surveillance period.

While prospective long-term data on WW patients followed for more than five years has not yet been reported, results from the OPRA trial and the International Watch and Wait Database suggest that very few treatment failures occur after 3 years of surveillance (59, 110). Longer follow-up is needed to determine precisely when patients can safely transition to survivorship care. Although not yet used in clinical practice, circulating tumor DNA may offer another method of surveillance, with increasing levels indicating possible local regrowth or distant metastases (111).

## 6 Local regrowth

Patients with local regrowth harbor residual disease not clearly evident during the restaging assessment. Between 15% to 36% of patients who enter WW surveillance develop local regrowth, with most cases occurring within two years of restaging (10, 11, 59, 112–115). Data from the International Watch and Wait Database indicate that 97% of all regrowths occur within the bowel wall, highlighting the importance of careful endoscopic surveillance (9). Unlike local recurrence following TME, where less than a third of patients have resectable disease, the vast majority of regrowths are salvageable (8, 11, 113).

The effects of local regrowth on oncologic outcomes remain poorly described and are difficult to determine using retrospective data. Studies suggest that patients with local regrowth have a higher risk of distant metastases compared to those with a s-cCR (11, 116) but have similar survival outcomes to patients with an iCR taken to TME immediately after restaging (59). However, local regrowths are a biologically distinct set of tumors with an excellent (but incomplete) response to neoadjuvant therapy. Therefore, patients with complete eradication of disease (s-cCR) and those with a poor response to treatment (iCR) cannot act as appropriate control groups.

Additionally, it remains unclear whether the delay in definitive surgical resection for patients with local regrowth increases the risk of distant metastases. Our understanding of the temporal relationship between metastatic progression and local regrowth is limited. Distant metastases may be a consequence of inherently poor tumor biology already present at diagnosis or may develop after TNT from occult cancer cells left at the primary tumor site (11, 116). Unfortunately, it is difficult to provide a clear answer to this question, as evaluating the impact of delaying surgery would require prospective randomized control trials that are unlikely to accrue. Current focus lies in accurate detection of regrowths with surveillance exams and an expedient TME once residual disease is suspected.

## 7 Watch and wait outcomes

Definitive proof of the safety and efficacy of nonoperative management requires a large phase III trial with a non-inferiority design. However, randomized control trials comparing selective WW and mandatory TME strategies are considered infeasible due to the risk of patient crossover into the WW arm. Until recently, most evidence supporting the safety of a WW strategy came from studies using multi-institutional, retrospective databases. However, these analyses could not account for differences in treatment regimens, clinical response criteria, timing of restaging assessment or surveillance protocols. The OPRA trial was the first prospective phase II trial to demonstrate that patients with an excellent response to TNT could achieve long-term organ preservation with oncologic outcomes equivalent to historical controls treated with the standard of care (chemoradiation, TME and adjuvant chemotherapy) (8). While the OPRA trial provides the best information to date on the safety and efficacy of nonoperative management, its findings are limited by constraints in study design. The trial did not randomize patients by treatment strategy (TME vs. WW) and instead relied upon comparisons to historical controls treated with a different neoadjuvant regimen (chemoradiation vs. TNT). Despite these limitations, the OPRA trial considerably improved our understanding of patient selection for WW and strengthened available evidence demonstrating that nonoperative management is a viable treatment option for patients with a complete response following TNT.

### 7.1 Long-term oncologic outcomes

The OPRA trial found similar rates of disease-free survival, distant metastasis-free survival, local recurrence-free survival and overall survival between patients treated with a selective WW strategy and historical controls treated with mandatory TME (59). Oncologic outcomes did not differ between patients who received INCT-CRT or CRT-CNCT (**Table 2**) (59) Given that historical controls were treated with chemoradiation while patients in the OPRA trial received TNT, it is possible that the benefits from upfront chemotherapy offset the potentially adverse oncologic effects of nonoperative management. However, the results of the OPRA trial are consistent with survival outcomes reported in the TNT arms of the PRODIGE-23, CAO/ARO/AIO-12, RAPIDO and GCR-3 trials (43–45, 47, 59, 117).

**Table 2:** Five-year survival outcomes from the OPRA trial.

Outcome	INCT-CRT (%)	CRT-CNCT (%)	P-value
Disease free survival	71	69	0.68
Local recurrence free survival	94	90	0.35
Distant metastasis free survival	80	78	0.64
Overall survival	88	85	0.25

Five-year survival outcomes reported in the OPRA trial (59). INCT-CRT, induction chemotherapy followed by chemoradiation; CRT-CNCT, chemoradiation followed by consolidation chemotherapy.

### 7.2 Organ preservation

The rate of organ preservation reported in the OPRA trial was higher than rates of pCR in historical controls treated with TNT followed by TME (3, 43, 44, 47, 59). This difference likely reflects the OPRA trial’s duration of neoadjuvant treatment, extended interval (8 ± 4 weeks) between end of TNT and restaging, and broad selection criteria permitting patients with a nCR to enter WW. A higher proportion of patients who achieved a s-cCR were randomized to the CRT-CNCT arm (54% vs. 39%; p=0.012) (59). Our understanding of why patients receiving CRT-CNCT have higher rates of organ preservation remains limited. Although the CAO/ARO/AIO-12 trial did not offer patients WW, the study demonstrated a similar pattern with higher rates of pCR in the CRT-CNCT group (47). While tumors treated with CRT-CNCT have a longer time-interval to regress between (chemo)radiation and restaging, this does not explain the differences in s-cCR observed in the OPRA trial. Patients in the INCT-CRT arm had a higher incidence of local regrowth, which may partially account for the lower rates of organ preservation in this group (8, 59). However, explanations of why INCT-CRT may predispose patients to an increased risk of local regrowth remain purely speculative.

Secondary analyses of the OPRA trial have demonstrated higher rates of organ preservation among patients with a cCR at restaging compared to those with a nCR (118). These differences are due to local regrowth, highlighting that patients with a nCR are high-risk for harboring residual disease and should be followed at short intervals if offered WW surveillance.

### 7.3 Quality of life

We would expect improved quality of life among WW patients compared to those undergoing TME, as nonoperative management preserves the rectum and patients have no postoperative recovery. However, very little information on this topic exists and the long-term effects of various multimodal neoadjuvant treatment regimens remain unclear. A retrospective case-control study by Quezada-Diaz et al. found that WW patients had better short-term bowel function compared to matched controls that underwent TME (119). A prospective study of 278 patients in the Dutch Watch and Wait Registry provides additional insight into long-term quality of life outcomes (120). The authors found that approximately 25% of WW patients have major low anterior resection syndrome (LARS) at 24 months from restaging, which is roughly half that reported in historical controls with a LAR (120, 121). Both male and female patients also reported significant rates of sexual dysfunction (120). These findings highlight that neoadjuvant treatment continues to have long-term consequences in the absence of surgical resection. Additional data from prospective studies comparing quality of life and functional outcomes between TME and WW patients is needed to better understand potential differences between the two groups.

## 8 Conclusions

WW has become an accepted alternative to TME in LARC patients with a complete response to neoadjuvant treatment. The OPRA trial demonstrated that over 45% of patients achieve long-term organ preservation with oncologic outcomes comparable to historical controls. While approximately 1/3 of patients who entered WW experienced local regrowth, all were salvageable by TME and this subgroup exhibited similar outcomes to patients with an iCR after TNT. Several challenges to expanding the use of WW remain. First, high-quality prospective data on the feasibility and oncologic safety of this strategy is limited to a single, phase II randomized control trial. Second, the restaging exam’s diagnostic performance continues to suffer from sub-optimal accuracy, particularly in identifying the presence of viable tumor cells that later become local regrowth. Third, the advantages of various TNT regimens with regards to maximizing a complete response, decreasing risk of local regrowth and minimizing treatment-associated toxicity remain unknown.

Future directions for research involve tailoring treatment regimens to optimize patient outcomes. This includes identifying genetic and molecular markers responsive to immunotherapy or predictive of a complete response (60, 122, 123). The Janus Rectal Cancer Trial, which randomizes patients to receive CRT-CNCT with doublet (FOLFOX or CapeOx) or triplet (mFOLFIRINOX) chemotherapy will provide data on the potential efficacy of a triplet chemotherapy regimen in improving rates of disease-free survival and organ preservation (124). Machine learning and radiomics may prove useful in improving the accuracy of restaging endoscopy and MRI (125, 126). Finally, WW remains limited to stage II or III rectal cancers. The ongoing STAR-TREC trial aims to evaluate the suitability of this approach for stage I disease treated with neoadjuvant (chemo)radiation (127).
